# Supplementary material for: Effective Prognostic Model for Therapy Response Prediction in Acute Myeloid Leukemia Patients
Source: J Pers Med. 2023 Aug 7;13(8):1234. doi: 10.3390/jpm13081234 (PMC10455213; doi:10.3390/jpm13081234)
Supplement: Supplementary file 1 [file jpm-13-01234-s001.zip › jpm-2507193-supplementary.pdf]

Table S1. Age distribution of AML patients (n=53).

Age, years	Abs.	%
18-19	1	1.9
20-29	4	7.5
30-39	7	13.2
40-49	7	13.2
50-59	18	34
60-69	12	22.6
70-79	4	7.6
Total	53	100

Table S2. Gender distribution of AML patients (n=53).

Total number of patients		Male		Female	
Abs.	%	Abs.	%	Abs.	%
53	100	25	47.2	28	52.8

Table S3. Distribution of AML patients according the presence of lymphadenopathy (n=53).

Lymphadenopathy	Total		Men		Women	
	Abs.	%	Abs.	%	Abs.	%
Presence of lymphadenopathy	9	17	4	7.6	5	9.4
Absence of lymphadenopathy	44	83	21	39.6	23	43.4
Total	53	100	25	47.2	28	52.8

Table S4. Distribution of AML patients according the presence and structure of extranodal diseases (n=53).

Extranodal diseases	Abs.	%
Presence of extranodal diseases	36	67.9
Liver	30	56.6
Lungs	5	9.4
Skin	1	1.9

Absence of extranodal diseases	17	32.1
Total	53	100

Table S5. The structure of clinical syndromes in AML patients (n=53).

Clinical syndrome	Abs.	%
Hemorrhagic	42	79.2
Anemic	50	94.3
Infectious	27	50.9
Hyperplastic	35	66
Intoxication	46	86.8
Total	53	100

Table S6. Parameters of peripheral blood in AML patients before treatment start (n=53).

Parameter (reference values)	MEAN ± SEM	Presence in patients	
		Abs.	%
Normal hemoglobin (Hb = 120-170 g/L)	143.3±27	3	5.6
Mild anemia (Hb = 120-90 g/L)	101.2±7.3	18	34
Moderate anemia (Hb = 90-70 g/L)	80.1±6.5	18	34
Severe anemia (Hb < 70 g/L)	55.9±8.5	14	26.3
Normal platelets, 10^9/L (150-400 × 10^9/L)	213.6±59.4	7	13.2
Thrombocytosis (platelets > 400 × 10^9/L)	549±9.9	2	3.8
Thrombocytopenia (platelets < 150 × 10^9/L)	35.1±28.8	44	83
Normal leukocytes (4-9 × 10^9/L)	6±1.5	7	13.2
Leukopenia (leukocytes < 4 × 10^9/L)	2±0.5	8	15.1
Leukocytosis (leukocytes 9-100 × 10^9/L)	44.3±26.4	26	49.1
Hyperleukocytosis (leukocytes > 100 × 10^9/L)	182.4±119.5	12	22.6
Blasts, %	55.7±29.3	42	79.2

Table S7. AML risk stratification by genetics*. Patient distribution (n=43).

Risk category	Genetic abnormality	Total number		Male		Female	
		Abs	%	Abs	%	Abs	%
Favorable	t(8;21)(q22;q22.1) / *RUNX1-RUNX1T1*<br>inv(16)(p13.1q22) or t(16;16)(p13.1;q22) / *CBFB-MYH11*<br>Mutated *NPM1* without *FLT3-ITD*<br>Mutated *CEBPA* (double)	23	53.5	15	34.9	8	18.6
Intermediate	Mutated *NPM1* with *FLT3-ITD*<br>Wild-type *NPM1* with *FLT3-ITD* (without adverse-risk genetic lesions)<br>t(9;11)(p21.3;q23.3) / *MLLT3-KMT2A*<br>Cytogenetic and/or molecular abnormalities not classified as favorable or adverse	5	11.6	2	4.6	3	7
Adverse	t(6;9)(p23;q34.1) / *DEK-NUP214*<br>t(v;11q23.3) / *KMT2A* rearranged<br>t(9;22)(q34.1;q11.2) / *BCR-ABL1*<br>inv(3)(q21.3q26.2) or t(3;3)(q21.3;q26.2) / *GATA2, MECOM(EVI1)*<br>-5 or del(5q); -7; -17/abn(17p)<br>Complex karyotype, monosomal karyotype<br>Mutated *ASXL1*, *BCOR*, *EZH2*, *RUNX1*, *SF33B1*, *SRSF2*, *STAG2*, *U2AF1* and/or *ZRSR2*<br>Mutated *TP53*	15	34.9	5	11.6	10	23.3
Total		43	100	22	51.1	21	48.9

*2022 European LeukemiaNet risk stratification by genetics [1].

1. Döhner, H.; Wei, A.H.; Appelbaum, F.R.; Craddock, C.; DiNardo, C.D.; Dombret, H.; Ebert, B.L.; Fenaux, P.; Godley, L.A.; Hasserjian, R.P.; et al. Diagnosis and management of AML in adults: 2022 recommendations from an international expert panel on behalf of the ELN. *Blood* 2022, *140*, 1345–1377, doi:10.1182/BLOOD.2022016867.

Table S8. AML prognosis stratification by cytogenetic/molecular markers and clinical characteristics. Patient distribution (n=53).

Prognosis category	Cytogenetic markers	Molecular markers	Clinical factors	Total number		Male		Female	
				Abs	%	Abs	%	Abs	%
Favorable	t(8;21)(q22;q22)<br>inv(16)(p13.1;q22) or t(16;16)(p13.1;q22)<br>t(15;17)	Mutated *CEBPA* (double)<br>Mutated *NPM1* (without *FLT3*-ITD mutation) with normal karyotype	MRD-negative	10	18.9	5	9.4	5	9.4
Unfavorable	inv(3)(q21;q26.2) or t(3;3)(q21;q26.2)<br>t(9;22)<br>t(9;11)<br>t(v;11)(v;q23)<br>t(6;9)(p23;q34)<br>-5 or del(5q)<br>-7<br>abn(17p)<br>Complex karyotype<br>Monosomal karyotype	Enhanced *Evi-1* expression<br>*MLL* rearrangements<br>*FLT3*-ITD mutation<br>*DNMT3A* mutation<br>*BAALC* expression<br>*ERG* expression<br>*MN1* expression<br>*WT1* polymorphism<br>*BCR-ABL*-positive<br>c-kit mutation for t(8;21) inv 16	Secondary AML<br>Increased age (>60 years; for inv(16) >35 years)<br>Elevated WBC count (>100×10^9/L; for t(8;21) >20×10^9/L)<br>Extramedullary disease<br>No early complete remission<br>Persistent MRD<br>$CD34^+$ blasts<br>M0 according FAB classification	43	81.1	20	37.7	23	43.4
Total				53	100	25	47.2	28	52.8

MRD – measurable residual disease

Table S9. Distribution of AML patients according to the therapy response after 1-2 courses of chemotherapy (n=53).

Therapy response	Total		Men		Women	
	Abs.	%	Abs.	%	Abs.	%
Complete remission	16	30.2	7	13.2	9	17
Relapsed disease	5	9.4	1	1.9	4	7.5
Primary chemoresistance (refractory disease)	32	60.4	17	32.1	15	28.3
Total	53	100	25	47.2	28	52.8
